# ‘Why should I worry, since I have healthy feet?’ A qualitative study exploring barriers to use of footwear among rural community members in northern Ethiopia

**DOI:** 10.1136/bmjopen-2015-010354

**Published:** 2016-03-22

**Authors:** Abebe Kelemework, Abebayehu Tora, Tsigie Amberbir, Getnet Agedew, Abiyu Asmamaw, Kebede Deribe, Gail Davey

**Affiliations:** 1 International Orthodox Christian Charities (IOCC), Debre Markos, Ethiopia; 2 Department of Sociology, Wolaita Sodo University, Sodo, Ethiopia; 3 Department of Sociology, Addis Ababa University, Addis Ababa, Ethiopia; 4 Department of Amharic Language, Literature Folklore, Addis Ababa University, Addis Ababa, Ethiopia; 5 Department of Ethiopian Languages and Literature, Debre Markos University, Debre Markos, Ethiopia; 6 Wellcome Trust Brighton and Sussex Centre for Global Health Research, Brighton, UK; 7 School of Public Health, Addis Ababa University, Addis Ababa, Ethiopia

**Keywords:** Podoconiosis, Footwear, Personal protection, Shoe wearing, Neglected tropical diseases

## Abstract

**Objective:**

To explore the influence of personal, cultural and socioeconomic factors related to footwear use and non-use in northern Ethiopia.

**Design:**

A qualitative study was conducted using focus group discussions and in-depth individual interviews. Data were collected using semistructured interview guides.

**Setting:**

The study was conducted in East and West Gojjam Zones, Amhara region, northwest Ethiopia.

**Participants:**

A total of 91 individuals from 4 target groups participated in individual and group interviews: (1) non-affected community leaders including *Idir* (a form of social insurance) leaders, school principals, *kebele* (the lowest administrative unit) officials, health professionals, teachers, merchants and religious leaders; (2) affected men and women; (3) non-affected men and women not in leadership positions; and (4) school children (both male and female).

**Results:**

Participants perceived a range of health benefits from donning footwear, including protection against injury and cold. Various types of shoes are available within the community, and their use varied depending on the nature of activities and the season. Personal and socioeconomic barriers hindered the desire to consistently use footwear. Widely established barefoot traditions and beliefs that footwear is uncomfortable, heavy and may weaken the feet have made the regular use of footwear uncommon. Economic constraints were also mentioned as hindering ownership and use of footwear. Distance from places where shoes could be bought also contributed to limited access. Cultural influences promoting gender inequality resulted in women being least able to access shoes.

**Conclusions:**

We identified several individual, cultural and socioeconomic barriers that influence individuals’ decisions about and use of footwear in rural northern Ethiopia. Promoting education on the health benefits of footwear, curbing podoconiosis-related misconceptions and integrating these with economic empowerment programmes, may all improve the use of footwear.

**Strengths and limitations of this study**

- A strength of this study is that it included a wide range of participants and so gained a variety of perspectives.
- Several barriers to shoe-wearing not previously identified emerged from this study.
- All participants were from rural northern Ethiopia, so the findings may not be generalisable beyond this.

## Introduction

Footwear can provide considerable health benefits in reducing the incidence or progression of a range of neglected tropical diseases (NTDs).^1^ Walking barefoot has long been considered an important risk factor for podoconiosis, a non-filarial, geochemical form of lymphoedema and elephantiasis that results in bilateral swelling of the lower legs.^2^ Podoconiosis imposes huge economic burdens that worsen the prevailing poverty, and results in considerable social stigmatisation associated with the belief that the condition is familial and incurable.^3^ ^4^ Available evidence indicates that podoconiosis is preventable if individuals consistently use footwear and begin doing so early in life.^5–8^ Footwear is also associated with low prevalence of other soil-transmitted diseases,^8^ and has been shown to prevent the recurrence of attacks of adenolymphangitis among patients with lymphoedema^9^ and to reduce foot complications in other diseases such as diabetes.^10^

Nevertheless, several studies have documented that a considerable proportion of rural communities do not use footwear, or use it only during specific seasons such as the rainy or hot seasons.^5^ ^7^ Inconsistent use of footwear is associated with increased risk of acquiring soil-transmitted and foot-related disease.^1^ ^8^ ^11–15^

People living in areas endemic for podoconiosis hold misconceptions about the causes of podoconiosis. Individuals believe, for example, that one may acquire podoconiosis through bad fortune, evil spirits or stepping on goat's blood.^4^ ^16–19^ Many people, studied in southern Ethiopia, believed that heredity made podoconiosis inevitable, and considered the presence of an inherited gene to be an absolute guarantee of podoconiosis occurrence. These beliefs were associated with decreased likelihood of preventive behaviours such as footwear use.^17^ ^19^

A study conducted in southern Ethiopia identified financial constraints, unsuitability of shoes for specific activities and low perception of risk, as barriers to footwear use.^5^ However, little is known about the personal, cultural and socioeconomic contexts of footwear use in northern Ethiopia, where podoconiosis is highly endemic in a population group with different cultural norms to those in southern Ethiopia. This study, therefore, aimed to explore the influence of personal, cultural and socioeconomic factors related to footwear use and non-use in northern Ethiopia.

## Methods

### Study area

The study was conducted in East and West Gojjam Zones, Amhara region, northwest Ethiopia. According to the 2007 census, East and West Gojjam Zones have a population of 4 260 533 people with an annual growth rate of 2.5%.^20^ The main economic activity in the zones is subsistence agriculture. The point prevalence of podoconiosis in East and West Gojjam Zones was estimated to be 3.4% in 2012.^18^

The International Orthodox Christian Charities (IOCC), a non-government organisation, launched the first podoconiosis prevention and treatment programme in East Gojjam Zone, Amhara Regional state, in 2010. The prevention and treatment package includes foot care and hygiene, raising awareness about the disease and rehabilitation of patients. Shoes donated by TOMS (a US-based shoe company that donates one pair of shoes for each pair sold) have been distributed to rural school children to reduce the risk of acquiring podoconiosis.

### Sampling and data collection

A qualitative study was conducted in August and September, 2014. Six of thirteen IOCC treatment sites were selected purposively based on their geographic representation and history of treatment services. A total of 91 individuals from four target groups participated: (1) non-affected community leaders: *Idir* (a form of social insurance) leaders, school principals, *kebele* (the lowest administrative unit) officials, health professionals, teachers, merchants and religious leaders; (2) affected men and women; (3) non-affected men and women not in leadership positions; and (4) school children (both male and female). A total of six focus group discussions (FGDs) were carried out: women's and men's group discussions were held separately for affected and unaffected people, whereas all community leaders joined one group. In each group, 8–12 individuals participated while 17 individual in-depth interviews (IDIs) were held with affected and unaffected individuals.

Semistructured interview guides adapted from those used in a similar study in southern Ethiopia^6^ were used to collect data, and additional items were included as data collection progressed. Data collection continued until saturation was reached. All interviews were conducted in *Amharic*, the native language of the local people, and recorded with consent. Interviews lasted on average 1 h for IDIs and 2 h for FGDs.

### Data coding and analysis

AA (Amharic language expert and native speaker) transcribed the data and collaborated with AT during translation. Three of the team members (AT, AA and AK) coded the data by themes predefined in the interview guides and emerging during analysis, using a grounded theory approach, a qualitative research methodology in which a researcher systematically identifies themes and concepts emerging from a chunk of text data and theorises, as coding is being carried out, about how each concept identified is related to a larger, inclusive concept.^21^ This was followed by reconciliation of coding by three members through frequent discussions on deviations and common themes. All team members were involved in draft organisation of codes and corresponding quotes to identify consistencies and contradictions in the data and interpretation. NVivo software for qualitative data analysis was used along with a manual approach.

### Ethical statement

Introductory letters were obtained from East and West Gojam Zonal Health Departments and Woreda Health Offices. Oral informed consent was obtained from each study participant: participation in the study was voluntary and any information provided was kept confidential. Quoted information was anonymised during the analysis and reporting.

## Results

The following major themes and subthemes were identified as barriers related to footwear: (1) misconceptions about podoconiosis and inaccurate risk perceptions; (2) the barefoot tradition; (3) the perceived importance of shoes for special occasions; (4) gender inequality; (5) perceived poverty; and (6) the perceived inaccessibility of shoe markets.

### Misconceptions about podoconiosis and inaccurate risk perceptions

Affected and unaffected participants were asked whether they knew what causes podoconiosis and how to prevent it. This was to explore whether shoe wearing as a preventive health behaviour was central to community members’ conceptions of podoconiosis cause and prevention. Many had no idea what caused the disease and few associated it with prolonged exposure to irritant soil particles. Lack of shoe wearing was rarely mentioned as a cause of the disease in response to such questions. Speculations regarding podoconiosis causes were categorised under three subdomains and are discussed below.

#### Spiritual factors as a cause

Beliefs that evil spirits cause podoconiosis are very common. For the majority, stepping on things thrown down by someone who is jealous or hostile was believed to be a major cause of podoconiosis. This is a major source of fear, and stems from the suspicion that an acquaintance thinks or does evil things to damage one's health either physically or psychologically.

I got the disease when I was crossing the river. It was after four years of marriage. When I was crossing the river, I stepped on tetracycline that was put in a small scarf and tied together with ‘injera’ [a kind of pancake made from a grain, ‘teff’]. My feet began swelling after that and gradually advanced. (Affected FGD female, age 35 years)

#### Overlooking barefoot exposure to soil as a cause

Barefoot exposure to soil was rarely mentioned as the cause of podoconiosis. However, participants often mentioned environmental and other risk factors such as cold weather, ‘mich’ (wounds believed to be caused by taking off shoes during burning sun), ‘yekola mujele’ (lowland jiggers), using second-hand shoes, sharing water with affected people and stepping on waste such as cattle dung or farm residues.

I know some people with swollen feet. But, I don't exactly know the cause. They usually mention exposure to cold weather as a cause. (Unaffected IDI Male, age 21 years)

I got the disease because of ‘chamma-mich’. This open plastic shoe exposes to ‘mich’. The disease is caused by ‘mich’ related to wearing shoes. (Affected IDI male, age 56 years)

The habit of eating goat meat was the most commonly perceived and feared cause of podoconiosis among the participants.

I have never been worried about the disease as those who eat goat meat are believed to get the disease in our community. I have never eaten goat meat. There is an affected woman in our neighborhood. When she was asked why her feet swelled, she said, “I have never eaten goat meat. Unknowingly, I ate goat meat in my friend's house”. Since I heard this, I never eat goat meat. (Unaffected Student FGD female, age 15 years)

Since exposure to soil is not commonly considered to be linked to podoconiosis, few participants consider that footwear might prevent it. Many indicated their efforts to prevent podoconiosis in avoiding the environmental risk factors discussed above:

I try to protect my children by advising them not to wear the shoes or socks I have worn. I throw away my old shoes into the toilet fearing they may use them and get the disease. (Affected IDI male, age 38 years)

Though I am not sure, I think I got the disease because of contact with sweat from my affected husband. But now, to prevent my children, we don't share water for washing feet. I dump the water I used for washing my feet and wash the container carefully before my children use it for washing their feet. (Affected FGD female, age 40 years)

#### Inaccurate perception of hereditary risk of podoconiosis

Associating podoconiosis with heredity was common. Amharic terms, such as ‘endezer hono’, ‘be zer’ and ‘keziriya’, were used to explain the role of heredity in the local language. However, participants had little knowledge of what was inherited through the blood line. Some participants confused heredity and contagion, either of which could lead to podoconiosis clustering within affected families. Even in families in which two or more members or blood relatives were affected by podoconiosis, affected participants associated the cause of their foot condition to sharing of beds, water, shoes or tools used by other affected people in the family. Footwear was often seen as the culprit rather than as a way of preventing podoconiosis:

It was at the time of DERG regime. I bought ‘Keskis chamma’ from someone. I started wearing the shoes. Just after a month, my feet began swelling. I was shocked and said ‘wa, what is this?’ My parents said, ‘please throw away this shoes into the river’. I didn't throw it away, but left it in the house. My younger brother wore the shoes unknowingly. As a result, his feet also started to swell. (Affected FGD male, age 60 years)

### Barefoot tradition

Participants indicated that owning shoes does not guarantee they are worn, because of the established barefoot tradition. They said that, for the majority of community members, barefoot walking was easier than using shoes. The barefoot tradition is manifested in various ways: in perceptions that shoes are heavy, or that they weaken the feet or fall outside ‘standard’ dress and are therefore not prioritised.

People feel at ease when they walk barefoot. Some consider shoes to be heavy particularly in the mud. They think walking barefoot speeds up performance of any activity including running. (Affected, FGD male, age 55 years)

Most adult community members find going barefoot more efficient in use of time and energy. In slippery places, many participants thought that walking barefoot prevented them from slipping. Under conditions where fast running was required (eg, being chased by an enemy or an animal), they said that those with shoes could not escape. Going barefoot was perceived to have many advantages in rural areas:

People usually avoid shoe wearing as they are not well aware of its importance. They think that their feet would go weaker and weaker and can't resist challenges for the future.(Affected man, age 75 years)

There is an old story. Two persons were talking each other. One was wearing shoes while the other was not. The one with shoes asked the other one, “my brother, why didn't you wear shoes?’ The other one replied “why should I weaken my foot which will serve me in bad days?” You know, wearing shoes was not common in our tradition. (Affected, male FGD participant, age 60 years)

Shoes are commonly ‘forgotten’, because they are not considered part of ‘standard’ dress. The subconscious self-monitoring of shoe use may be affected by the lack of a shoe-wearing tradition in the community. When we challenged a person who claimed to wear shoes regularly but was without shoes at the time of interview, he said

I forgot to wear them as I was rushing to come here. (Unaffected IDI male, age 25 years)

The barefoot tradition results in shoes being given lower priority than clothes. Many community members would prefer to be without shoes than without clothes. They fear being considered ‘mad’ if they do not wear clothes, but the same is not a consequence of not wearing shoes.

I give priority to clothes. It depends on where you live. It is rare to see people using shoes in our community. People decorate themselves with clothes not shoes. Hence, if you want to be equal with others, you need to have clothes. It does not matter if you don't have shoes. And, if you appear with shoes all the time, people say ‘he is boastful’. People will laugh at you if they see you working in the farm with shoes. I never saw a person wearing shoes while plowing or cultivating the land. (Unaffected IDI male, age 21 years)

### Perceived importance of shoes for special occasions

The term ‘shoes’ is universally known among community members and it is rare to find a person who has never seen or heard of ‘shoes’ these days in rural areas in northern Ethiopia. Several local terms are available for different types of shoes. The most common Amharic word used is ‘chamma’. Participants could list various kinds of footwear available in their community, each given different names. For instance, open shoes made locally from tires are called ‘barbasso’, ‘yegebere chama’ (farmer's shoes), ‘ekedeke’, ‘gelet’, etc. ‘Gomma chamma’ is a common term for dry rubber plastic shoes while ‘kofkuafie’ refers to foam-type rubber plastic shoes. ‘Shera chamma’ refers to canvas shoes, while ‘koda chamma’ are shoes made of leather. ‘Bot chamma’ is a common name for both dry and foam rubber plastic boots while ‘keskis chamma’ is used to refer to boots made from leather, particularly for soldiers.

Participants relate different types of shoes to different activities and seasons. They adapt their use of shoes according to the situation.

I have three types of shoes. I use ‘yegebere chamma’ [shoes made from tyres] whenever I travel far away or for working on the farm in the dry season, to protect my feet from injuries. Compared to other shoes, they withstand hardships and can be used for about 7 to 8 years. (Unaffected IDI male, age 21 years)

The types of farming activities influence the pattern of shoe use in several ways. For instance, during threshing and clearing weeds from the farm, shoes are not worn. The major reason for not wearing shoes while clearing weeds is so as not to damage crops:

when we enter into the farm with shoes, it damages the plants. (Unaffected IDI male age 21 years)

The reason for not wearing shoes during threshing is harder to rationalise, but is deep set in community traditions.

it is taboo to wear shoe at ‘beray’ [threshing season] time…you know why?…it is a sign of respect to the crops. (Unaffected IDI male, age 28 years)

If an individual lacks appropriate shoes for a task or season, he or she is likely to go barefoot. For instance, ‘barbasso’ shoes are impractical during ‘kiremt’ (the rainy season).

In the rainy seasons, ‘barbasso’ is less functional in muddy and slippery places as it potentially exposes to injuries related to slipping, and it does not protect the feet from cold weather since it does not cover them entirely. I have ‘eke-deke’. But, I didn't wear them today as it is so muddy. So, I went out barefoot because I cannot walk with shoes in the mud. I use ‘eke-deke’ in dry time. As it does not fully cover the foot, mud enters into it. (Unaffected FGD male, age 53 years)

Similarly, closed rubber plastic footwear, whether short shoes or boots, are commonly worn in the rainy season as they are thought to protect the feet from the cold. In the hot season, plastic boots are avoided because of the discomfort and bad smell associated with them. Participants also felt that plastic boots were not comfortable for long journeys.

It is difficult to use plastic boots for long journeys. When it is hot, they collect blood from our body and make it clot around our feet. Also, when it's hot, plastic boots cause a bad smell. But they are helpful for cold weather and mud since they are light. (Unaffected FGD male, age 35 years)

Canvas shoes are preferred during the dry and cold seasons and by young people. Overall, community members are less likely to own or wear them as they become dirty and are easily damaged as they trap dust and mud.

I like canvas shoes. Others of my age also prefer canvas or sneaker shoes. I feel discomfort wearing plastic boots or leather shoes because they are heavy. Whenever I go to market or for holidays, I use canvas shoes. I wear plastic sandals when I am around home. But, the problem is, you cannot wear canvas shoes in the rainy season because of dirt and mud. (Unaffected IDI male, age 21 years)

Leather shoes, particularly those that are closed, seem to be the least owned or worn type of shoes in rural areas for several reasons. First, the perceived cost of leather shoes is very high. Second, they are thought to be very heavy. Third, they are suitable for the hot or rainy seasons.

I don't have leather shoes, but my father has. My foot size and his are similar. When I check his shoes, even the soles are heavy. You can wear canvas shoes without socks. But you cannot comfortably wear leather shoes without socks. When socks are added, they become heavier. If I need to walk about two to three hours on foot to go to ‘Merawi’ [name of nearby town], it is really heavy. As a result I don't like to buy leather shoes. But, they are also so expensive even when you need it. (FGD student male, age 18 years)

Some participants thought that, unlike other types of shoes, leather shoes require socks and washing of the feet whenever they are worn. Washing the feet and socks every time before and after using closed leather shoes is impossible for rural farmers, whose lives are associated with mud and dust every day.

I bought new leather shoes. When I wore them without socks, they hurt the skin around the toes. I then started using socks. It increased the comfort. But, it brought a bad smell to my feet. I took off the socks and washed my feet. And I started using ‘kongo’ plastic shoes. If you wear leather shoes, it requires washing feet and socks immediately you use them. (Unaffected FGD male, age 35 years)

Some participants linked the difficulties of wearing shoes that require socks and foot washing with chronic shortages of water in their locality:

In the dry season, we face serious shortages of water, even for drinking. There is no tap water. There is one spring where everyone goes there. We wait overnight to get it. (Unaffected FGD male, age 59 years).

### Gender inequality

Discussions held with all categories of participants revealed that both the pattern of shoe use and the types of preferred shoes differed between women and men. Men are relatively advantaged in terms of availability of shoes appropriate for various activities, and have a lighter burden of day-to-day tasks. In addition to farm work, women shoulder the burdens of carrying out domestic chores and trading in the market. As a result, it is common to observe men wearing shoes while they go to market, while most women are barefoot and burdened by many other items.

If the road is muddy, men take off their shoes, hang them on their stick and carry them across their shoulders. But, as women already carry things on their back, they cannot handle shoes. They are physically weak compared to males. While carrying things on their back, they cannot walk wearing shoes in the muddy and bumpy roads. The shoe adds weights to the heavy things they carry on their back. (Affected FGD male, age 55 years)

It is men who frequently wear shoes. We carry many things when we go to market or other places. Men do not carry anything when they go to distant places. We carry ‘tela’ [local beverage], bread, ‘injera’, etc. Since shoes are heavy, we take them off and walk barefoot. (Unaffected FGD female, age 50 years)

The types of shoes that are locally available also favour men. Boots (both leather and plastic) and ‘barabasso’ are more commonly worn by men. Though some women wear plastic boots, many consider that doing so would expose them to ridicule. It is rare for women to wear ‘barabasso’ simply because the community is accustomed to seeing men wearing them. If a woman wears shoes and neat clothes all the time, she may be pointed at or gossiped over—people will speculate that she is looking for another partner or has a foot problem like ‘mujale’ (jiggers) or leprosy. It is not only men who ridicule women who wear shoes regularly; women also take part.

For instance, if the girl appears with shoes during a wedding ceremony, people say “her feet are deformed due to ‘mujale’” or suspect other problems. They say, “Why does she wear shoes if her feet are clean?” There are also other conditions; those women who wear shoes regularly are demeaned. (Affected IDI female, age 25 years)

Shoes were thought not to be a priority for many women, compared with clothes and ‘timtam’ (a scarf tied around the head). Compared with men, many women own shoes but do not wear them, except for special occasions. Wedding gifts are a good example: the groom is expected to buy a ‘kemis’ (skirt), ‘ambar’ (bracelet) and ‘timtam’ for the bride, while the groom's family buys him special shoes.

My brother is married. After he participated in the Community Conversation conducted by IOCC, he wears shoes all the time. Though he bought two pairs of shoes for his wife, she does not like to wear them. When I told her to wear shoes when she goes somewhere, she said, “why should I worry since I have healthy feet”. She left the shoes in her house. (Affected IDI female, age 25 years)

### Perceived poverty

Most participants said that poverty was a major deterrent to ownership and use of footwear. They admitted that shortage of money meant they did not always own or wear shoes, often saving them for special occasions rather than wearing them out in everyday activities.

In fact, shoes are very expensive for poor people. Those who don't have assets, find it so difficult to buy shoes. These days, the cost of shoes is 300 to 400 [Birr, US$15–20] even for canvas shoes, let alone leather shoes. Life is very expensive nowadays. (Affected FGD male, age 52 years)

### Inaccessibility of shoe market

Shoes are often unavailable in smaller village markets. Traders supply better quality shoes to the larger markets, usually in towns. Rural residents, particularly those in remote villages, may have to either walk for several hours on foot or use a vehicle to get to market, which may also limit their motivation to purchase shoes.

We go to Debremarkos [the Zonal capital] to buy shoes. There is a small market in Robit, very near, but shoes are not supplied there. We usually walk on foot to go to Debremarkos. We find a vehicle once in a week that is on Saturday. Saturday is the largest market day so that many people go to Debremarkos. Other times, we walk on foot. On average, it takes 3 to 4 hours to walk. (Unaffected IDI male, age 21 years)

## Discussion

This study explored the barriers to footwear use in an area endemic for podoconiosis and several NTDs. We identified individual behaviours and structural factors that influence individuals’ decisions on footwear ownership and use. We found that misconceptions about podoconiosis and inaccurate risk perceptions, barefoot traditions, the perceived importance of shoes for special occasions, gender inequality, perceived poverty and perceived inaccessibility of shoe markets, are the most important barriers to the ownership and use of footwear among rural community members in northern Ethiopia.

Underestimating the importance of barefoot exposure (due to misconceptions about podoconiosis and inaccurate risk perceptions) was found to be an important barrier to the use of footwear. This was echoed in previous studies in podoconiosis-endemic settings.^1^ ^2^ ^5–8^ Existing barefoot traditions were other reasons that individuals avoided wearing shoes. Beliefs that being barefoot is advantageous and that wearing shoes is potentially dangerous still prevail in the community. The barefoot tradition also results in shoes being afforded lower priority than clothes. This finding is congruent with a study in southern Ethiopia,^5^ but quite different to those of studies investigating other health commodities such as bed nets. Utilisation of bed nets was found in 73% and 68.6% of households in malaria-endemic areas (Arba Minch Town and Raya Alamata district, respectively^22^ ^23^), suggesting considerably greater priority given to bed nets in those areas than shoes in this area.

Though community members report that they own certain types of shoes, the perception that shoes are only for special occasions has been a major barrier to their use. The use of shoes also varies with the type of shoe owned, the activities being undertaken and the season. As shoes are believed to damage the crops, people do not wear shoes while entering into the field. It is also considered taboo to wear shoes while threshing crops. Choices of shoe type are made according to the weather, for instance, ‘Barbasso’ shoes are avoided during ‘kiremt’ (rainy season). However, closed rubber plastic footwear, whether short shoes or boots, are commonly worn in the rainy season, as they are believed to protect the feet from cold weather. During the hot season, plastic boots are avoided due to the discomfort and the bad smell associated with them. Interestingly, heat was also given as a reason for 15.7% of households not using bed nets in Raya Alamata.^22^

Gender inequality is the most important determinant of optimum use of shoes. In many instances, men are relatively advantaged due to a lower burden of day-to-day tasks, meaning that they are able to wear shoes more frequently than women. Consequently, unlike males, females usually go to market barefoot, since they think that shoes will be difficult to carry in addition to their other burdens. The types of shoes that are locally available are most appropriate for men. Most shoe types are worn by men, and if a woman appears in shoes she is likely to be ridiculed, pointed at or gossiped over by the community (both males and females). An earlier study in the same study setting demonstrated more men than women to be wearing shoes at the time of interview,^18^ as did a study in western Ethiopia.^15^ Recent national podoconiosis mapping showed that more men than women were wearing shoes at the time of interview.^24^ Overall gender mainstreaming and women's empowerment might address this challenge in the long term. In the short term, however, it will be important to address this gap during free shoe distribution for the prevention of podoconiosis. As for communities in southern Ethiopia,^5^ participants in this study confirmed that poverty was another barrier to the ownership and use of footwear, as is the inaccessibility of shoe markets.

## Conclusion

Although the nature of this qualitative study may limit its generalisability to other contexts, it reveals information that is likely to be helpful in guiding further research and interventions to prevent podoconiosis. Using footwear optimally to prevent multiple NTDs is contingent on addressing these barriers. Increasing community awareness about the causes of NTDs, social transformation to diminish barefoot traditions and to create favourable attitudes towards footwear, increasing access to affordable footwear and addressing broader gender inequalities, are recommended to increase the wider acceptance and utilisation of footwear as an important public health intervention. The Ethiopian government may also consider adopting a policy of footwear use in schools, as is now the case in certain other African countries.
